# Multiscale dynamic modeling and simulation of a biorefinery

**DOI:** 10.1002/bit.27099

**Published:** 2019-07-21

**Authors:** Tobias Ploch, Xiao Zhao, Jonathan Hüser, Eric von Lieres, Ralf Hannemann‐Tamás, Uwe Naumann, Wolfgang Wiechert, Alexander Mitsos, Stephan Noack

**Affiliations:** ^1^ Process Systems Engineering (AVT.SVT) RWTH Aachen University Aachen Germany; ^2^ Institute of Bio‐ und Geosciences, IBG‐1: Biotechnology Forschungszentrum Jülich GmbH Jülich Germany; ^3^ Software and Tools for Computational Engineering RWTH Aachen University Aachen Germany; ^4^ Bioeconomy Science Center (BioSC) Forschungszentrum Jülich GmbH Jülich Germany; ^5^ Computational Systems Biotechnology (AVT.CSB) RWTH Aachen University Aachen Germany

**Keywords:** biorefinery, DAEO, DFBA, dynamic modeling, Modelica

## Abstract

A biorefinery comprises a variety of process steps to synthesize products from sustainable natural resources. Dynamic plant‐wide simulation enhances the process understanding, leads to improved cost efficiency and enables model‐based operation and control. It is thereby important for an increased competitiveness to conventional processes. To this end, we developed a Modelica library with replaceable building blocks that allow dynamic modeling of an entire biorefinery. For the microbial conversion step, we built on the dynamic flux balance analysis (DFBA) approach to formulate process models for the simulation of cellular metabolism under changing environmental conditions. The resulting system of differential‐algebraic equations with embedded optimization criteria (DAEO) is solved by a tailor‐made toolbox. In summary, our modeling framework comprises three major pillars: A Modelica library of dynamic unit operations, an easy‐to‐use interface to formulate DFBA process models and a DAEO toolbox that allows simulation with standard environments based on the Modelica modeling language. A biorefinery model for dynamic simulation of the OrganoCat pretreatment process and microbial conversion of the resulting feedstock by *Corynebacterium glutamicum* serves as case study to demonstrate its practical relevance.

## 1 INTRODUCTION

Biorefineries are a promising approach to produce chemicals and fuels from sustainable natural resources to reduce carbon dioxide emissions and the dependency on fossil raw materials. Techniques from computer‐aided process design play an important role in increasing competitiveness of bio‐based processes compared with existing conventional processes (Bao, Ng, Tay, Jiménez‐Gutiérrez, & El‐Halwagi, 2011; Kokossis & Yang, 2010; Martín & Grossmann, 2013).

Most conventional industrial processes have been studied and improved for decades resulting in highly integrated processes with near optimal cost and resource efficiency. Similar to conventional processes, a biorefinery comprises a variety of process steps, for example, biomass pretreatment, microbial transformation, and downstream processing, to convert bio‐based feedstock into desired products. A high integration of these process steps and the capability to produce multiple products is crucial for a competitive biorefinery (Kokossis & Yang, 2010). This in turn requires plant‐wide biorefinery modeling as an important tool at different stages of process development.

During early‐stage process design, a large number of product and process alternatives is analyzed and compared with respect to economic and environmental criteria using mathematical optimization. Simple models are used, for example, yield coefficients for reactions (Bao et al., 2011; Voll & Marquardt, 2012) and short‐cut methods for separation steps (Ulonska, Skiborowski, Mitsos, & Viell, 2016), combined with detailed models (e.g., equilibrium and kinetic models) where data are available (Kelloway & Daoutidis, 2014; Martín & Grossmann, 2013) to identify promising feedstocks and reaction pathways. More detailed, steady‐state, plant‐wide process models are used to investigate and improve individual biorefinery concepts. For example, Tay, Kheireddine, Ng, El‐Halwagi, and Tan (2011) investigated gasification of lignocellulosic biomass in an integrated biorefinery using a superstructure optimization framework with models of different complexity.

In the next step, these biorefinery concepts are experimentally investigated in pilot plants requiring dynamic process models to improve process understanding and to allow model‐based process operation and control. As the number of considered process alternatives decreases, more detailed models can be formulated. While this is true for most process steps, the complexity of microbial transformations is usually not represented on the same level of detail. In the past, mostly unstructured (so called black‐box) models were used to approximate the reaction kinetics of microbial transformations.

However, this class of models is not suitable to describe and predict the complex intracellular metabolism of microorganisms cultivated in bioreactors for the purpose of bio‐based production. Clearly, to understand these multilevel interactions in a real quantitative manner, mechanistic pathway modeling in combination with multi‐omics analytics would be required (Wiechert & Noack, 2011). Although a couple of promising examples in this direction do exist (Gonçalves et al., 2017; Hameri, Fengos, Ataman, Miskovic, & Hatzimanikatis, 2018; Noack, Voges, Gätgens, & Wiechert, 2017; Zieringer & Takors, 2018) this approach is still hampered by the availability of in vivo enzyme kinetic data for relevant metabolic reactions.

As an intermediate solution, dynamic flux balance analysis (DFBA) enables the simulation of consecutive, steady‐state metabolic flux distributions under changing environmental conditions (Mahadevan, Edwards, & Doyle, 2002; Palsson, 2008; Zhao, Noack, Wiechert, & von Lieres, 2017). DFBA is based on a linear equation system covering the stoichiometry of all intracellular reactions within defined network boundaries and a differential equation system covering the time‐dependent behavior of extracellular biomass, substrate, and product concentration (Mahadevan et al., 2002). In most cases, the system of equations is under‐determined. Formulation of a cellular optimization criterion, for example, maximization of growth or ATP production, allows choosing among the set of solutions to determine one particular metabolic flux distribution. Finally, the DFBA model represents a system of ordinary differential equations with embedded optimization criteria (ODEO; Zhao et al., 2017).

Unfortunately, this type of mathematical problem is hard to solve and requires specialized solution methods that are commonly categorized as static optimization approach (SOA), dynamic optimization approach (DOA), and direct approach (DA). SOA divides the time horizon into intervals, solves the embedded optimization problem at the beginning of each interval and performs integration assuming a fixed flux distribution for the respective interval (Mahadevan et al., 2002). DOA discretizes the dynamic equations using collocation methods to obtain a nonlinear problem (NLP) that needs to be solved only once (Mahadevan et al., 2002). DA uses a solver to evaluate the embedded optimization problem in each time step during integration (Hanly & Henson, 2011; Hjersted & Henson, 2006; Zhuang et al., 2011). Höffner, Harwood, and Barton (2013) have implemented a DFBA simulator that uses first‐order optimality conditions to reformulate the embedded optimization problem into a system of differential‐algebraic equations (DAEs).

Based on these solution techniques, the DFBA approach was successfully used for various applications, for example, for the investigation of the diauxic growth of *Escherichia coli* on d‐glucose (Mahadevan et al., 2002), the optimization of a fed‐batch fermentation process with *Saccharomyces cerevisiae* on d‐glucose (Hjersted & Henson, 2006), and the optimization of co‐cultures with both model organisms on mixtures of d‐glucose and d‐xylose (Hanly & Henson, 2011).

One particular advantage of the DFBA approach is its ability to account for changing environmental conditions during (fed‐)batch operation. Therefore, we chose the multiscale modeling approach by embedding a DFBA model into a plantwide biorefinery simulation. Mathematically, this leads to a system of differential‐algebraic equations with embedded optimization criteria (DAEO).

In this study, we present a framework that enables dynamic plant‐wide biorefinery modeling and simulation. The framework builds on Modelica as powerful equation‐based modeling language and specific libraries for easy implementation of different submodels describing biomass treatment and microbial transformation processes. The integration of a detailed DFBA model into a biorefinery design process enables conclusions regarding the interaction of individual process steps, design of potential feedback loops, and development of model‐based control systems. To demonstrate the practical relevance of the approach a two‐step biorefinery model is implemented, describing the dynamics of the OrganoCat pretreatment process and the microbial conversion of lignocellulose‐derived sugars by the model organism *Corynebacterium glutamicum*.

## 2 METHODS

### 2.1 Modelica library for biorefinery modeling

We implemented a library of dynamic models for unit operations that are widely used in chemical processes, for example, flash unit, decanter, and distillation column, using the object‐oriented modeling language Modelica (Fritzson, 2015). Modelica is well‐suited for modeling biorefineries as it allows replacement of simple models with more detailed ones as new insights become available and also enables replacement of entire process steps if desired. The library uses the components Modelica.Fluid and Modelica.Media of the Modelica Standard Library (Casella, Otter, Proelss, Richter, & Tummescheit, 2006; Elmqvist, Tummescheit, & Otter, 2003). The former provides an interface for one‐ and zero‐dimensional physical modeling of thermo‐hydraulic components (Casella et al., 2006). The component equations (e.g., mass and energy balance equations) are decoupled from fluid property equations (e.g., calculation of specific enthalpy or density). In this way, the same component model can be used with different fluids (Casella et al., 2006). The Modelica.Media library defines the fluid and allows implementation of different models such as ideal gases and multiphase systems (Elmqvist et al., 2003).

In this study, we built on a similar structure compared with the open source libraries described above. Balance equations, for example, mass and energy balances for an ideally mixed phase, are decoupled from property calculation for the mixture. We formulated lumped models, that is, no spatial dependency of variables was considered. In dynamic modeling of relevant unit operations, there is still a large variety of modeling assumptions that is only covered to a certain extent, for example, by allowing the user to specify different degrees of freedom. For property calculation, we implemented relevant thermodynamic models in Modelica. These routines may be replaced by a suitable thermodynamic property package interfaced via the External Object utility of Modelica. The required parameter values were stored in a Modelica record that defines the medium (in contrast to a package in the Modelica.Media library). The parameter record also defines the models to be used for calculation of thermodynamic properties such as specific enthalpy or molar volume. The property equations were implemented as Modelica functions.

The respective unit operation model defines either single‐ or multiphase equations. For multiphase unit operations a quasi‐homogeneous modeling approach was used. Consequently, equilibrium was assumed for the respective phases. Examples for the most relevant phase equilibria and the associated unit operations are vapor‐liquid equilibrium (VLE) for flash unit and distillation column or liquid–liquid equilibrium (LLE) for decanter and extraction column. An equation‐oriented formulation for phase calculation that allows dynamic simulation in all phase regimes, that is, in single‐ and two‐phase region, was implemented based on literature for VLE (Biegler, 2010; Gopal & Biegler, 1999; Sahlodin, Watson, & Barton, 2016) and LLE (Müller & Marquardt, 1997; Ploch, Glass, Bremen, Hannemann‐Tamás, & Mitsos, 2019). The phase calculation may again be performed externally using a suitable package. Reactors were modeled with input–output relations based on experimental data or kinetic models if available. The piping between two building blocks was not considered, that is, energy and pressure loss were neglected. The graphical interface allows generation of process models at the flowsheet level.

### 2.2 DFBA process model for microbial transformation

The dynamic model for the microbial conversion step is based on the DFBA approach given by

(1a) $$\dot{x}\left(t\right)=f\left(x\left(t\right),v\left(t\right),p,t\right),\;\text{with}\;x\left(t=0\right)=\;x_{0},$$

(1b) $$v\left(t\right)\in \;\text{arg}\underset{\hat{v}}{\min}\,h\left(\hat{v}\right)$$

(1c) $$\text{s.t.}\;N\hat{v}=0$$

(1d) $$C\hat{v}⩽b\left(x\left(t\right),p,t\right),$$

(1e) $${\hat{v}}^{lb}⩽\hat{v}⩽{\hat{v}}^{ub}.$$

where x are the $n_{x}$ differential states describing the extracellular environment with initial conditions given by $x_{0}$. The differential equations f depend on the optimal solution v of an embedded optimization problem. Note that a typical DFBA process model does not include any algebraic equations outside the embedded optimization problem. Therefore, we refer to equation system (1a) as ODEO. The optimization problem is based on classical FBA. The stoichiometry of the reaction network is defined by the stoichiometric matrix N with dimensions $n_{m}\times n_{v}$ and the vector $\hat{v}$ covering $n_{v}$ reaction fluxes. The equations are derived from $n_{m}$ steady‐state mass balances for the $n_{m}$ intracellular metabolites assuming that the metabolite pools adjust infinitely fast to changing environmental conditions (Stephanopoulos, Aristidou, & Nielsen, 2008). Additional constraints on the reaction fluxes are imposed by Equation (1e), for example, to consider irreversibility assumptions for some fluxes. The dynamics of the extracellular environment define upper bounds for uptake rates of the intracellular reaction network (Equation (1d)).

For a typical biochemical network, the number of metabolic fluxes is larger than the number of metabolites ($n_{v}>n_{m}$), that is, the constrained linear system of equations given by (1c), (1d), and (1e) is under‐determined and a cellular optimization criterion is formulated to determine one particular flux distribution v. The objective functions used in literature are either linear, for example, maximization of cell growth (Hanly & Henson, 2011; Varma & Palsson, 1994) or ATP production (Raghunathan, Pérez‐Correa, Agosin, & Biegler, 2006) or nonlinear, for example, minimization of the overall intracellular flux (Schuetz, Kuepfer, & Sauer, 2007) or maximization of biomass yield per flux unit (Zhao et al., 2017). In this study, we used a linear objective function that can be defined by $h\left(\hat{v}\right)=-c^{T}\hat{v}$, where c is the cost vector.

## 3 RESULTS AND DISCUSSION

### 3.1 Framework for plant‐wide biorefinery modeling and simulation

**Figure 1:**
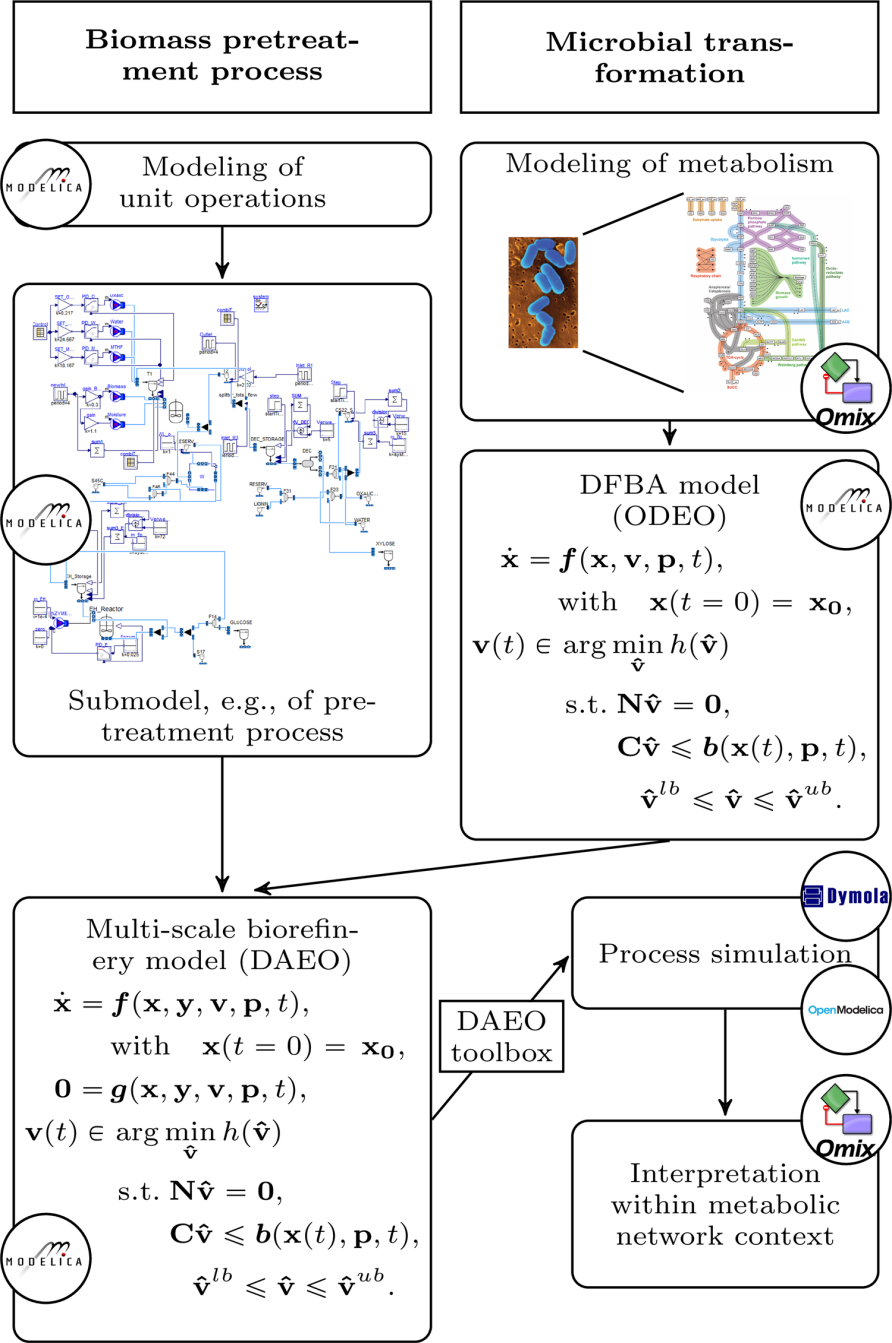
Framework for plant‐wide biorefinery modeling and simulation. For modeling biomass pretreatment processes a library of dynamic unit operations was implemented in Modelica. Thermodynamic property parameters were extracted from Aspen Plus and converted to a Modelica record. For modeling the microbial transformation step the visualization tool Omix is used and the resulting metabolic network is parsed to Modelica code. An external program interprets the code and creates the DFBA submodel. The external program applies the direct solution method via an interface to LP solver CPLEX. This way, a simple and easy‐to‐use interface is obtained and Dymola or OpenModelica can be used to solve the resulting multiscale biorefinery model (DAEO). The results can again be interpreted in Omix [Color figure can be viewed at wileyonlinelibrary.com]

We established a framework for dynamic modeling and simulation of biorefineries that covers several steps, which are illustrated in Figure 1 and briefly described in the following:

1. We implemented a model library of building blocks in Modelica enabling dynamic modeling of early process steps such as pretreatment of lignocellulosic biomass (see Methods section for more details).
2. For the microbial transformation step, we apply the visualization tool Omix (Droste, Miebach, Niedenführ, Wiechert, & Nöh, 2011) to construct the metabolic network model of interest. The resulting stoichiometric model is then translated into Modelica code and the embedded optimization problem is also formulated in Modelica.
3. The DFBA process model is obtained by coupling the embedded optimization problem to the extracellular environment of a continuous stirred‐tank reactor leading to a submodel for the microbial transformation step (see Methods section for more details).
4. The coupling of different biorefinery submodels, for example, biomass pretreatment and microbial transformation, leads to a multiscale biorefinery model. Mathematically, this model is a DAEO and a tailor‐made solution method is required to allow process simulation in standard software such as Dymola^1^ and OpenModelica^2^. We use the direct approach for solving the embedded optimization problem in an external program that is interfaced to the respective process simulation software (cf. Zhao et al., 2017, section 3.2).
5. Finally, simulation studies with a compiled version of the DAEO model can be performed in MATLAB^3^ and the results can again be interpreted in Omix.

The Modelica library, the Omix converter and the DAEO toolbox are available for interested users.

In the following we applied this framework to simulate the dynamics of the OrganoCat pretreatment process and the subsequent conversion of the resulting sugar streams by *Corynebacterium glutamicum* in different scenarios.

### 3.2 Dynamic model of OrganoCat process

Plant‐derived biomass as feedstock has to be pretreated to make the native biomolecules accessible for subsequent chemical or microbial transformation. The effectiveness of a pretreatment depends on many factors such as type of biomass, process conditions, formation of unwanted degradation products, recyclability of catalysts, energy and catalyst costs, and many more. An overview of various pretreatment processes for lignocellulosic biomass, their advantages and drawbacks as well as an economic assessment can be found elsewhere (Hendriks & Zeeman, 2009; Mosier et al., 2005).

In this study, we considered the OrganoCat process (Grande et al., 2015; Viell, Harwardt, Seiler, & Marquardt, 2013; Vom Stein et al., 2011) as an example process for pretreatment of lignocellulosic biomass. It uses a biphasic solvent system for selective depolymerization of lignocellulosic biomass into three separate process streams under mild reaction conditions (Vom Stein et al., 2011). Two of these process streams consist of a mixture of C5 (mainly d‐xylose) and C6 (mainly d‐glucose) sugars with defined ratios that are envisioned to be processed in microbial transformation steps to yield high‐value products. To discuss the impact of different sugar ratios we define the fraction of total carbon atoms stemming from d‐xylose as

(2) $$\phi_{\text{C5}}=\frac{5\;\cdot \;c_{\text{C5}}}{5\;\cdot \;c_{\text{C5}}+6\;\cdot \;c_{\text{C6}}}$$

with $c_{\text{C5}}$ and $c_{\text{C6}}$ denoting the concentrations of d‐xylose and d‐glucose, respectively. Note that $\phi_{\text{C5}}=0$ refers to a pure d‐glucose solution, while $\phi_{\text{C5}}=1$ indicates pure d‐xylose.

**Figure 2:**
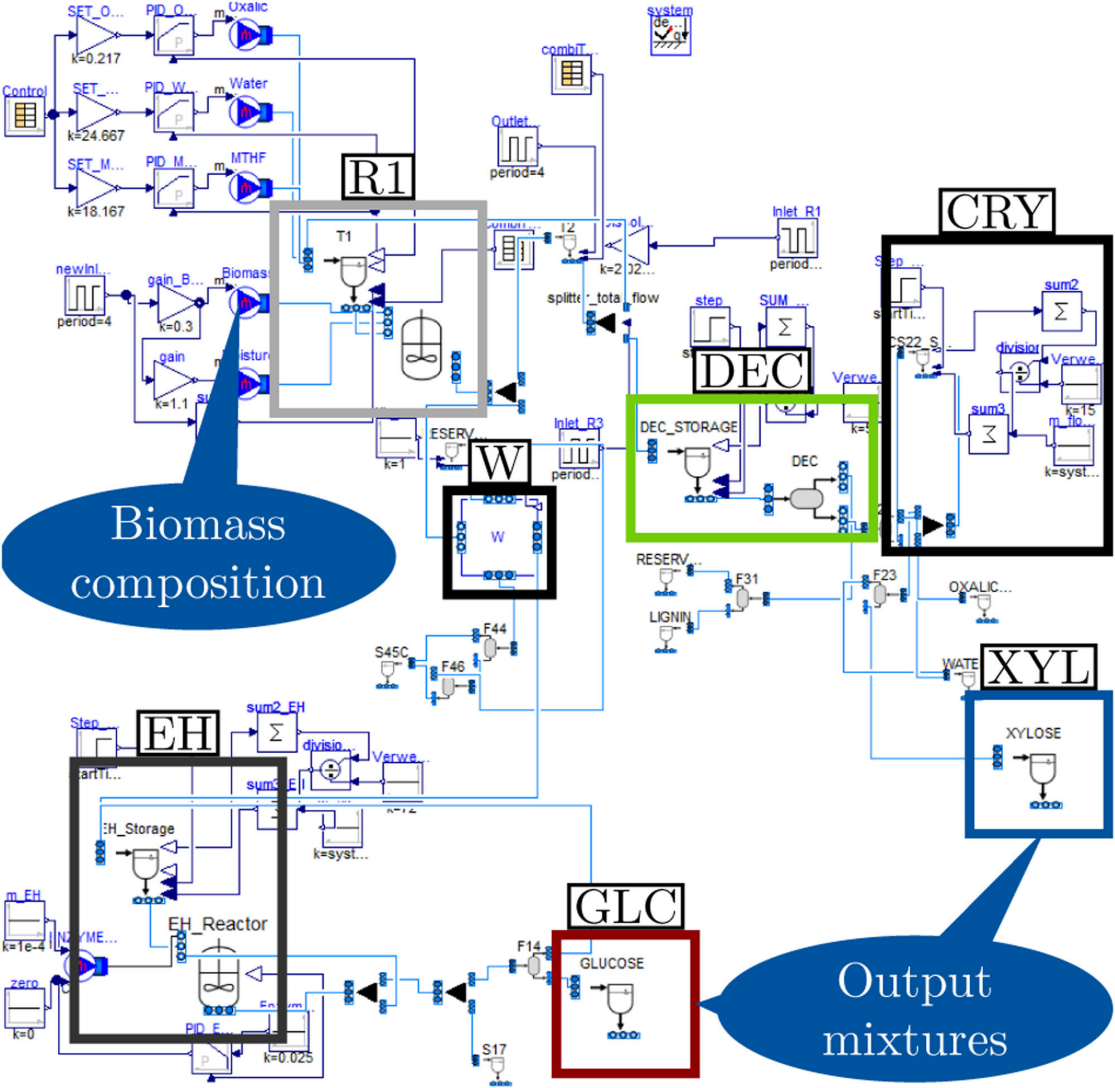
Dymola flowsheet of implemented OrganoCat pretreatment process. The biomass is exposed to a biphasic reaction medium (R1) to dissolve lignin and hemicellulosic sugars in the reaction medium. The cellulose fraction remains solid and is subsequently washed (W) and converted into a d‐glucose rich sugar stream (GLC) via enzymatic hydrolysis (EH). The liquid outlet of R1 is decanted (DEC) to obtain an organic fraction with dissolved lignin and a d‐xylose rich sugar stream (XYL) from the aqueous fraction. Crystallization (CRY) is used to recycle catalyst oxalic acid from the aqueous fraction [Color figure can be viewed at wileyonlinelibrary.com]

The dynamic model of the repetitive‐batch OrganoCat process that was formulated in this study is based on the conceptual process design of Viell et al. (2013) and the process variation proposed by Grande et al. (2015). Thermodynamic parameters were equal to the ones used in steady‐state simulation (Viell et al., 2013) except for the LLE between water and 2‐MTHF that was described by the NRTL model using up‐to‐date parameter values from literature (Glass, Aigner, Viell, Jupke, & Mitsos, 2017). By applying ideal dynamic unit operations and some additional assumptions (instantaneous flash units, residence time reactors for enzymatic hydrolysis and oxalic acid crystallization) a start‐up process was implemented (Figure 2).

**Figure 3:**
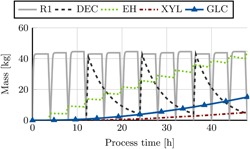
Simulation results for dynamic pretreatment process (repetitive‐batch OrganoCat). Hold‐ups of the respective unit operations (as indicated in Figure 2) are shown. The reactor R1 reuses the same medium three times before it is directed to a decanter (DEC). In between each cycle, the solid cellulose pulp is removed. It is further converted to monomeric sugars via enzymatic hydrolysis (EH) yielding a sugar stream with high d‐glucose content (GLC, $\phi_{\text{C5}}=0.09$). After decantation, the aqueous fraction yields a d‐xylose rich sugar stream (XYL, $\phi_{\text{C5}}=0.86$) [Color figure can be viewed at wileyonlinelibrary.com]

In the first process step, lignocellulosic biomass (e.g., beech wood) is exposed to a biphasic reaction medium consisting of water with oxalic acid and 2‐MTHF for a fixed reaction time in a batch reactor (Figure 2, R1). After the reaction time, the cellulose pulp is filtered off and further degenerated in subsequent process steps. The reactor is refilled with the biphasic reaction medium and fresh biomass is added. The mass of water, oxalic acid and 2‐MTHF is assumed constant in each cycle requiring addition of fresh reaction medium in between two cycles to compensate for the loss with regard to the removal of wetted cellulose pulp. The overall mass in the reactor increases from the first to the third cycle because of dissolved sugars in the reaction medium (Figure 3, R1). After the third cycle, the reaction medium is directed towards downstreaming and the reactor is filled with fresh reaction medium. Following the first biomass pretreatment step, the cellulose pulp contains sugars in solid form while another portion of sugars is already dissolved in the reaction medium. Both streams require further processing to obtain dissolved sugars in the right purity to serve as feedstock for microbial transformation.

A washing unit (Figure 2, W) is required to reduce the oxalic acid concentration of the solid cellulose pulp before enzymatic hydrolysis (Viell et al., 2013). For degradation of cellulose into monomeric sugars via enzymatic hydrolysis we use a reactor with a fixed average residence time of 72 hr (Figure 2, EH). After further purification (mostly instantaneous flash units), a stream with a defined sugar ratio of $\phi_{\text{C5}}=0.09$ is obtained (Figure 3) that is a potential feedstock for microbial transformation with a higher requirement on d‐glucose as carbon and energy source.

The liquid fraction is separated into an organic and aqueous phase in a continuously operated decanter (Figure 2 and Figure 3, DEC). After further purification steps (again mostly instantaneous flash units), the aqueous phase yields a sugar stream that has a greater portion of d‐xylose ($\phi_{\text{C5}}=0.86$, Figure 3) as it comprises the original hemicellulose fraction of the biomass (Figure 2, XYL). The oxalic acid is recycled from the organic phase via crystallization with an average residence time of 15 hr (Viell et al., 2013). Both the remaining aqueous and organic phase may be re‐used in the biphasic reactor.

### 3.3 Dynamic model of microbial transformation with *C. glutamicum*

For the microbial transformation step, we considered the platform organism *C. glutamicum* that is known for its capability to produce a variety of value‐added products including amino acids, organic acids, aromatic compounds, and proteins (Baritugo et al., 2018). Wild‐type *C. glutamicum* cannot naturally utilize d‐xylose, but in recent years the isomerase pathway and the Weimberg pathway as oxidative strategies for d‐xylose metabolization could be functionally implemented into this organism (Meiswinkel, Gopinath, Lindner, Nampoothiri, & Wendisch, 2013; Radek et al., 2014). Interestingly, there are two further alternative routes for d‐xylose assimilation known, namely, the oxido‐reductase and the DAHMS pathway (see, e.g., Valdehuesa et al., 2018, for a general overview on these pathways), but none of these has been tested for *C. glutamicum* so far.

**Figure 4:**
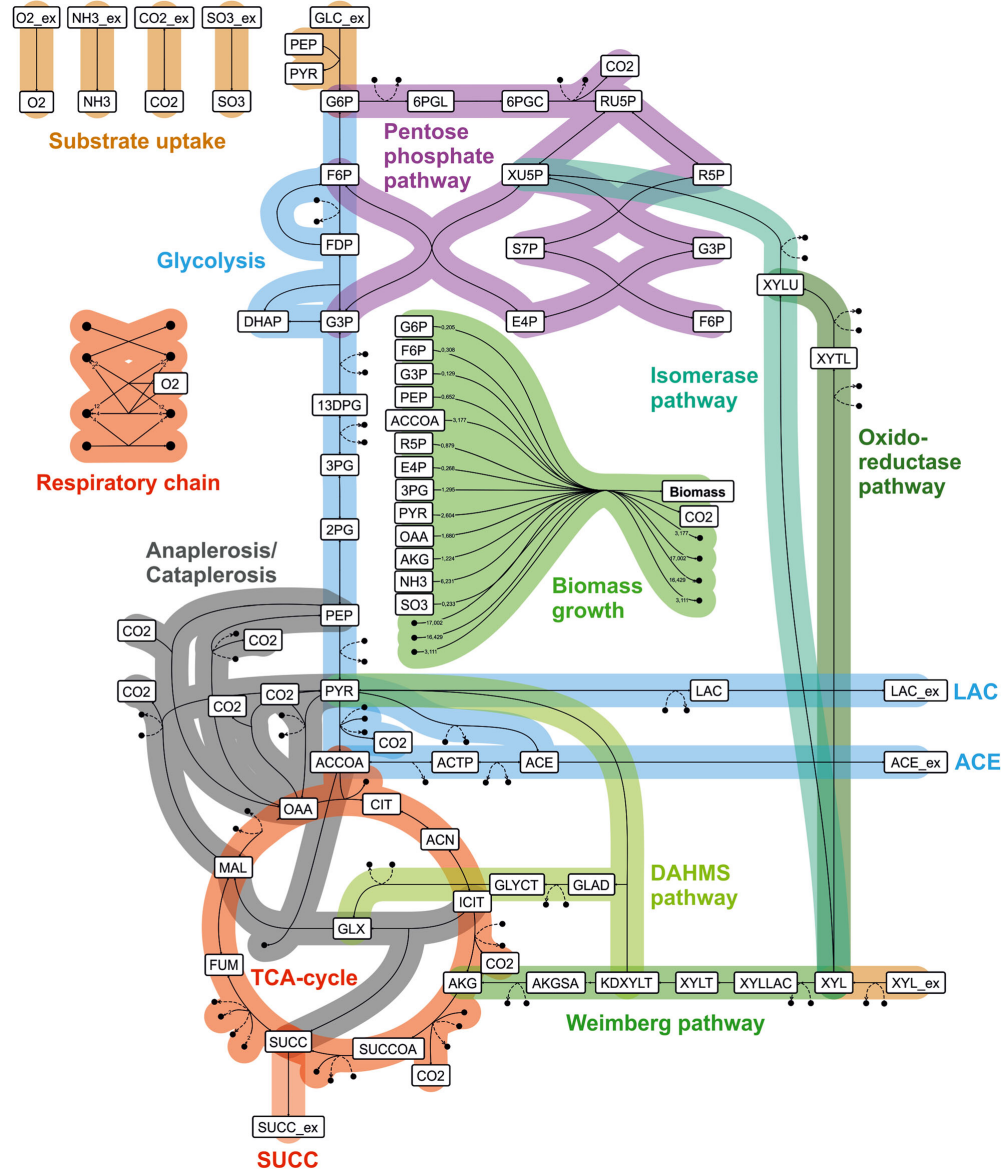
Omix representation of the central carbon metabolism of *Corynebacterium glutamicum* and additional catabolic routes for the pentose d‐xylose [Color figure can be viewed at wileyonlinelibrary.com]

Therefore we were interested to study each of these pathways under operation in *C. glutamicum* alone or in combination toward their potential to support the conversion of different OrganoCat media. For in silico analysis, a focused model of the central carbon metabolism of *C. glutamicum* was formulated and further extended with the four different d‐xylose metabolic pathways (Figure 4). Thereby, we are able to investigate the optimal assimilation pathways under different extracellular conditions as the solution of the embedded optimization problem decides upon their activity or inactivity. The model consists of 50 intracellular metabolites, 59 metabolic fluxes, and six additional exchange fluxes.

The balanced extracellular species are the carbon sources d‐glucose (GLC) and d‐xylose (XYL), the biomass (X) as well as the model target product succinate (SUCC) and by‐products lactate (LAC) and acetate (ACE). Uptake rates for d‐glucose and d‐xylose were modeled by classical Michaelis–Menten kinetics. In addition, an upper limit for the overall uptake rate of carbon sources was imposed to account for potential transport limitations:

(3) $$v_{\text{GLC}}+v_{\text{XYL}}⩽v_{\text{upt,max}}.$$

Maximization of biomass growth via the specific growth rate μ was used as optimization criterion in all simulations. The full DFBA model including kinetic parameter values is given in Appendix A.

#### 3.3.1 Case study: Growth of *C. glutamicum* on OrganoCat media

**Figure 5:**
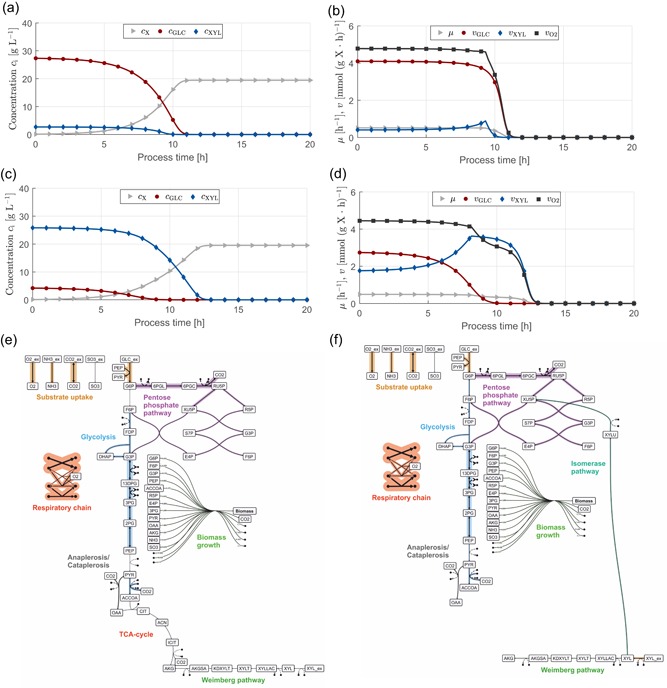
Model prediction for aerobic growth of *Corynebacterium glutamicum* on two different sugar streams from OrganoCat pretreatment. Extracellular concentration profiles and rates for biomass growth (X), as well as d‐glucose (GLC), d‐xylose (XYL), and oxygen ($O_{2}$) uptake are shown in the upper graphs. μ is the biomass growth rate. Further details on model parametrization are given in the Appendix (a). Concentration profiles for $\phi_{\text{C5}}=0.09$; (b) extracellular rates for $\phi_{\text{C5}}=0.09$; (c) concentration profiles for $\phi_{\text{C5}}=0.86$; (d) extracellular rates for $\phi_{\text{C5}}=0.86$; flux distributions for (e) $\phi_{\text{C5}}=0.09$ *t* = 1 and (f) $\phi_{\text{C5}}=0.86$, both at t=1 hr [Color figure can be viewed at wileyonlinelibrary.com]

In a first simulation study, we examined the potential of our advanced model strain *C. glutamicum* for aerobic growth on two different sugar streams as expected outcome of the OrganoCat pretreatment process (cf. Figure 2, GLC and XYL). The resulting profiles are shown in Figure 5. To simulate aerobic growth, the maximum oxygen uptake rate was not constrained, enabling an optimal oxygen uptake rate and maximum biomass growth under the changing substrate conditions.

Using the OrganoCat medium with high d‐glucose content ($\phi_{\text{C5}}=0.09$) as feedstock, biomass growth is achieved via simultaneous metabolization of both carbon sources without any by‐product formation (Figure 5a). The C5 sugar d‐xylose is depleted first after approximately 10 hr and biomass growth continues on pure d‐glucose. Up to the time of carbon source depletion, 19.9 g $L^{-1}$ biomass are formed. The uptake rate of d‐glucose is at the maximum possible value according to Michaelis–Menten kinetics indicating that d‐glucose is the preferred carbon sources (Figure 5b). d‐xylose is synthesized simultaneously with a lower uptake rate leading to a high overall growth rate of $\mu \approx 0.51\;{hr}^{-1}$. The oxygen uptake rate during growth is $v_{O_{2}}=4.78\;\text{mmol}\;{\left(g\;X\;\cdot \;hr\right)}^{-1}$. The decrease of d‐glucose concentration (after approx. 9 hr) leads to an increase of the d‐xylose uptake rate before its depletion. In Figure 5e, the metabolic flux distribution at t=1 hr is shown. Under these conditions, d‐xylose is expected to be exclusively metabolized via the Weimberg pathway to synthesize the essential biomass precursor α‐ketoglutarate.

For the other OrganoCat medium ($\phi_{\text{C5}}=0.86$), both carbon sources are again simultaneously consumed and similar amounts of biomass are formed (19.5 $g\;L^{-1}$, Figure 5c). This time, d‐glucose is depleted first because of the small initial amounts and the fast consumption. The small initial amount also leads to a smaller d‐glucose uptake rate in the beginning and, in turn, to higher d‐xylose uptake (Figure 5d). After depletion of d‐glucose, the organism continues growth on d‐xylose as sole carbon source. The flux distribution during initial growth phase shows that d‐xylose is metabolized via Weimberg and isomerase pathway (while oxido‐reductase and DAHMS pathway are not active, Figure 5f). The uptake via Weimberg pathway leads again to synthesis of growth precursor α‐ketoglutarate (with a rate, that is, required for optimal growth) resulting in a high growth rate of $\mu =0.49\;{hr}^{-1}$. Compared with growth on higher d‐glucose contents, no additional d‐glucose is channeled into this precursor. After depletion of d‐glucose, the sole carbon source d‐xylose is metabolized similarly via Weimberg and isomerase pathway ($\mu =0.34\;{hr}^{-1}$).

The simulation results indicate that growth on both OrganoCat media yields almost equal biomass titer. The space‐time yield is higher for $\phi_{\text{C5}}=0.09$ because the higher content of the preferred carbon source d‐glucose leads to a higher growth rate. Consequently, growth on this medium is preferable from a process perspective.

#### 3.3.2 Case study: Impact of varying sugar ratios on *C. glutamicum* growth

Following this outcome, we performed a second simulation study to investigate the influence of different sugar ratios on cell growth under aerobic batch operation. The main objective during growth in batch operation is high biomass titer in short batch time, that is, maximization of space‐time yield with respect to biomass defined by ${\text{STY}}_{X}=\frac{c_{X}\left(t_{B}\right)}{t_{B}}$, where $t_{B}$ is the final batch time defined by depletion of carbon sources and $c_{X}\left(t_{B}\right)$ is the biomass concentration at that timepoint. The maximum oxygen uptake rate was again not constrained to model aerobic growth conditions. In this scenario we looked into different strain designs regarding the activity of the four different d‐xylose metabolic pathways. In the first design, all of the four different pathways are potentially available and the solution of the optimization problem decides upon activity or inactivity of the respective pathway. In the other designs, only one of the four metabolic pathways is considered to be active by constraining the corresponding other reaction fluxes to be zero.

**Figure 6:**
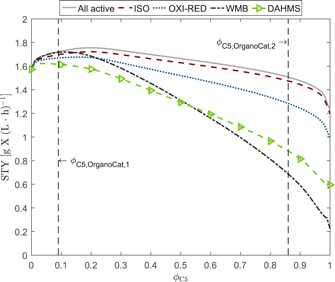
Prediction of space‐time yields for biomass of *Corynebacterium glutamicum* when grown in batch mode and varying mixtures of d‐glucose and d‐xylose. The set‐up for the simulation study is as follows: A batch reactor (V=1 L) with aerobic growth conditions, a constant inoculum concentration ($c_{X}\left(t_{0}\right)=0.1\;g\;L^{-1}$), and a constant overall amount of carbon atoms was simulated for different values of $\phi_{\text{C5}}\left(t_{0}\right)$ defining the initial ratio of C5 and C6 sugars (cf. Equation (2)). Vertical dashed lines indicate the two potential mixtures derived from OrganoCat pretreatment process (cf. Figure 2) [Color figure can be viewed at wileyonlinelibrary.com]

The results of this simulation study indicate that the STY is higher for growth on pure d‐glucose ($\phi_{\text{C5}}=0$) than on pure d‐xylose ($\phi_{\text{C5}}=1$), independent on the activity or inactivity of the different d‐xylose assimilation pathways (Figure 6). However, the highest STY is found for mixtures of both carbon sources because higher overall substrate uptake rates are obtained resulting in higher overall growth rates. For the strain design where all pathways may potentially be active (solid line, Figure 6), the highest STY is found at $\phi_{\text{C5}}=0.2$. Noteworthy, for a small content of C5 sugars ($\phi_{\text{C5}}⩽0.05$), d‐xylose is exclusively metabolized via the Weimberg pathway to synthesize biomass growth precursor α‐ketoglutarate (dash‐dotted line, Figure 6). This also holds true for increasing d‐xylose content, that is, the oxidative part of the TCA cycle is still inactive and surplus of d‐xylose is metabolized via the isomerase pathway. For the single pathway designs and higher d‐xylose content ($\phi_{\text{C5}}>0.15$) the operation of the isomerase pathway leads to significantly higher space‐time yields compared to all other pathways. This can be explained by the favorable energetics because reducing equivalents such as NAD(H) and NADP(H) are better balanced.

#### 3.3.3 Case study: Production of succinate with *C. glutamicum* under microaerobic conditions

In our third simulation study, we were interested in an optimal production scenario for succinate under microaerobic fed‐batch conditions. Therefore we coupled the optimal media design for biomass growth in a first batch phase (i.e., $\phi_{\text{C5}}=0.2$, cf. Figure 6) with a subsequent fed‐batch phase under conditions of varying oxygen availability. The degrees of freedom in this study are the sugar ratio $\phi_{\text{C5,in}}$ in the feed and the maximum oxygen uptake rate $v_{O_{2},\text{max}}$ during the feeding phase. Noteworthy, during feeding phase, substrates may accumulate in the reactor. To facilitate the comparison of different conditions, the process simulation was terminated when carbon sources were depleted (i.e., simulation is continued without feeding until threshold is reached). We define the space‐time yield with respect to the fed‐batch phase according to ${\text{STY}}_{\text{SUCC}}=\frac{c_{\text{SUCC}}\left(t_{F}\right)}{\Delta t_{\text{FB}}}$, where $t_{F}$ is the final process time, $c_{\text{SUCC}}\left(t_{F}\right)$ the concentration of succinate at that timepoint and $\Delta t_{\text{FB}}$ the period of the feeding phase (i.e., 45 hr) plus the duration until all carbon sources were depleted.

**Figure 7:**
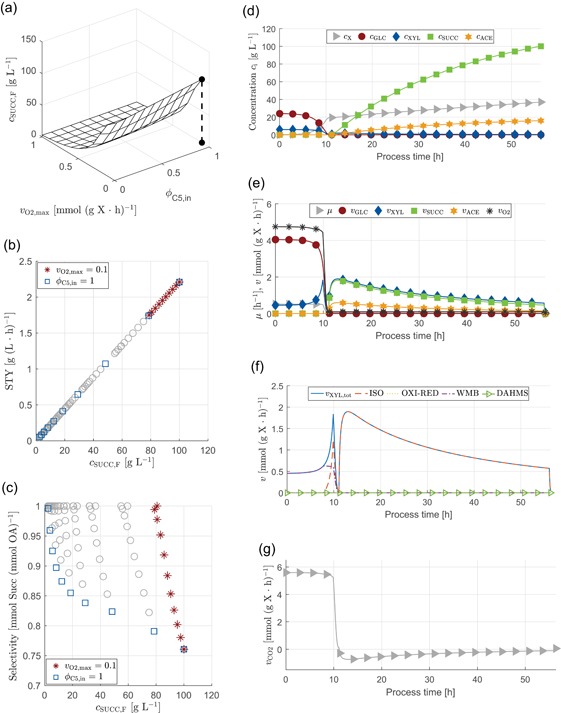
Prediction of the impact of sugar ratios $\phi_{\text{C5,in}}$ and oxygen availability $v_{O_{2},\text{max}}$ on succinate production with *Corynebacterium glutamicum* under microaerobic fed‐batch conditions in terms of final titer, space‐time yield and selectivity (left‐hand side) and model prediction for combination of optimal batch and fed‐batch operation for highest product titer (right‐hand side). The set‐up for the simulation study was as follows: Constant volumetric feeding rate of $F_{\text{in}}=0.02\;L\;{hr}^{-1}$, fixed inlet concentration of carbon atoms of 10 mol $L^{-1}$, variation of sugar ratio $\phi_{\text{C5,in}}$ in feed, and oxygen availability via the flux $v_{O_{2},\text{max}}$ [Color figure can be viewed at wileyonlinelibrary.com]

As a result, the highest product concentration of $100\;g\;L^{-1}$ is reached for production on a feed of pure d‐xylose and lowest oxygen availability ($v_{O_{2},\text{max}}=0.1$, Figure 7, left side). The trade‐off between space‐time yield and final product titer shows linear behavior, because only small amounts of sugars accumulate (due to low feed concentration and high biomass titer) leading to similar overall production period $\Delta t_{\text{FB}}$ in all cases.

The highest product titer, however, comes at low selectivity (amount of succinate per total amount of carbonic acids formed) due to simultaneous formation of acetate. Consequently, based on the assumed strain design (i.e., *C. glutamicum* wild type harboring four potentially active d‐xylose assimilation pathways, but no further strain modification, Figure 4) microaerobic production of succinate always leads to a trade‐off between high product titer and high selectivity.

The combination of optimal batch and fed‐batch operation with highest product titer is shown on the right‐hand side of Figure 7. During the batch phase (0⩽t⩽11 hr), a high growth rate is obtained by metabolization of d‐glucose at the highest possible rate. In addition, d‐xylose is metabolized via the Weimberg pathway to synthesize biomass precursor α‐ketoglutarate. Both carbon sources are depleted almost at the same time. During production phase, pure d‐xylose is fed at a low concentration such that only small amounts accumulate in the reactor. d‐xylose is metabolized via the isomerase pathway while all other pathways are inactive. Finally, oxygen limitation enforces the downregulation of the oxidative part of the TCA cycle and reverse operation of its reductive part leading to combined formation of succinate and acetate.

## 4 CONCLUSION AND OUTLOOK

We presented a multiscale and multidisciplinary modeling framework that allows dynamic modeling and simulation of complex biorefinery processes. The developed Modelica library of (bio)chemical unit operations permits easy adaptation to describe different subprocesses for the conversion of renewable feedstock into value‐added products or to replace (sub)models when new insights and more detailed models become available. The DFBA approach enables realistic modeling and simulation of microbial transformation steps and accounts for changing environmental conditions during dynamic operation. Our solution strategy for the embedded optimization problem is easy to use and allows problem formulation and modification in Modelica language. Finally, our case study showed that sugar mixtures from OrganoCat pretreatment are well‐suited carbon sources for bio‐based production with the platform organism *C. glutamicum*.

The direct approach is suitable for solving the embedded optimization problem during dynamic simulation of DFBA models. However, it is important to note that numerical difficulties may arise because active set changes of the embedded optimization problem are hidden from the integrator. In addition, the direct solution approach requires the simulation software to use a numerical approximation technique (e.g., finite differences) to obtain derivative information for the embedded optimization problem inducing an truncation error. For the case studies in this contribution, none of these difficulties were observed. Nevertheless, a more advanced solution method to handle these drawbacks is desirable. The method of Höffner et al. (2013) determines the active set of the embedded optimization problem and uses first‐order optimality conditions for reformulation into an algebraic equation system. Active set changes are indicated by zero‐crossings that can be detected by the integrator if coupled with a suitable event detection algorithm. Exact derivatives are obtained by using algorithmic differentiation techniques on the algebraic equation system (Naumann, 2012). Algorithmic differentiation is also beneficial for sensitivity analysis if an upper‐level optimization problem is considered, that is, in context of parameter estimation, optimal experimental design or optimal control.

The promising simulation results presented in this study need to be confirmed experimentally and the biorefinery model may be extended with respect to downstream processing to allow plant‐wide evaluation of the different production scenarios discussed in this study. In addition, the substitution of some (sub)models of the pretreatment process by more detailed models is necessary for in‐depth analysis of the influence between the various process steps and identification of reasonable feedback loops. For instance, a more detailed model of the enzymatic hydrolysis step would enable a feedback loop regarding the required sugar ratios for microbial transformation and appears to be a promising handle to improve the overall process performance. In addition, the presented tool may be used to support scale‐up of biorefinery processes, for instance, in terms of plant‐wide model‐predictive control, sizing of equipment, and identification of potential bottlenecks (e.g., availability of reactants, important recycle streams, accumulation of potential inhibitors for enzymatic hydrolysis, or microbial transformation).

## CONFLICT OF INTERESTS

All authors declare no competing interests.

## AUTHOR CONTRIBUTIONS

T. P. implemented the Modelica models and the DAEO toolbox, conducted the case studies, and prepared the manuscript. X. Z. implemented the interface between OMIX and Modelica. J. H. helped to finalize the manuscript. Ev. L. guided the research and edited the manuscript. R. H. implemented the parser of the DAEO toolbox and conceived the research. U. N. reviewed and improved the manuscript. W. W. guided the research and edited the manuscript. A. M. conceived the research, defined the case study and edited the manuscript. S. N. created the cell model, guided the research and helped to prepare the manuscript. All authors read and approved the submitted manuscript.

## DATA AVAILABILITY

The Modelica library, the Omix converter, and the DAEO toolbox will be made available for interested users.

## Footnotes

1 Dymola, 2018

2
https://www.openmodelica.org/

3 MATLAB R2017b

## APPENDIX A DFBA MODEL

The DFBA model to describe growth of *Corynebacterium glutamicum* comprises the following equations

(A1a) $$\dot{V}=F_{\text{in}},\;V\left(t_{0}\right)=V_{0},$$

(A1b) $${\dot{c}}_{\text{GLC}}=\frac{F_{\text{in}}}{V}\,\cdot \left(c_{\text{GLC},\text{in}}-c_{\text{GLC}}\right)-v_{\text{GLC}}\,\cdot c_{X},\;c_{\text{GLC}}\left(t_{0}\right)=c_{\text{GLC},0},$$

(A1c) $${\dot{c}}_{\text{XYL}}=\frac{F_{\text{in}}}{V}\,\cdot \left(c_{\text{XYL},\text{in}}-c_{\text{XYL}}\right)-v_{\text{XYL}}\,\cdot c_{X},\;c_{\text{XYL}}\left(t_{0}\right)=c_{\text{XYL},0},$$

(A1d) $${\dot{c}}_{X}=v_{X}\,\cdot c_{X}-\frac{F_{\text{in}}}{V}\,\cdot c_{X},\;c_{X}\left(t_{0}\right)=c_{X,0},$$

(A1e) $${\dot{c}}_{\text{SUCC}}=v_{\text{SUCC}}\,\cdot c_{\text{X}}-\frac{F_{\text{in}}}{V}\,\cdot c_{\text{SUCC}},\;c_{\text{SUCC}}\left(t_{0}\right)=c_{\text{SUCC},0},$$

(A1f) $${\dot{c}}_{\text{LAC}}=v_{\text{LAC}}\,\cdot c_{\text{X}}-\frac{F_{\text{in}}}{V}\,\cdot c_{\text{LAC}},\;c_{\text{LAC}}\left(t_{0}\right)=c_{\text{LAC},0},$$

(A1g) $${\dot{c}}_{\text{ACE}}=v_{\text{ACE}}\,\cdot c_{\text{X}}-\frac{F_{\text{in}}}{V}\,\cdot c_{\text{ACE}},\;c_{\text{ACE}}\left(t_{0}\right)=c_{\text{ACE},0},$$

(A1h) $$v\left(t\right)\in \text{arg}\min_{\hat{v}}-c^{T}\hat{v}$$

(A1i) $$\text{s.t.}\;N\hat{v}=0,$$

(A1j) $${\hat{v}}_{\text{GLC}}⩽v_{\text{GLC},\text{max}}\,\cdot \frac{c_{\text{GLC}}}{c_{\text{GLC}}+K_{\text{M},\text{GLC}}},$$

(A1k) $${\hat{v}}_{\text{XYL}}⩽v_{\text{XYL},\text{max}}\,\cdot \frac{c_{\text{XYL}}}{c_{\text{XYL}}+K_{\text{M},\text{XYL}}},$$

(A1l) $${\hat{v}}_{\text{GLC}}+{\hat{v}}_{\text{XYL}}⩽v_{\text{upt,max}},$$

(A1m) $${\hat{v}}_{{\text{O}}_{2}}⩽v_{{\text{O}}_{2},\text{max}},$$

(A1n) $${\hat{v}}^{lb}⩽\hat{v}⩽{\hat{v}}^{ub}.$$

Here, the differential variables are the reaction volume V and extracellular concentrations $c_{\text{GLC}}$, $c_{\text{XYL}}$, $c_{\text{X}}$, $c_{\text{SUCC}}$, $c_{\text{LAC}}$ and $c_{\text{ACE}}$ of d‐glucose (GLC), d‐xylose (XYL), biomass (X), succinate (SUCC), lactate (LAC), and acetate (ACE) with differential equations (A1a) to (A1g). The extracellular balance equations are coupled to the embedded optimization problem (A1h)–(A1m) via growth rate, uptake and excretion kinetics. The uptake kinetics are described by equations (A1j) and (A1k) where the upper bound is given by Michaelis–Menten kinetics with parameters $v_{\text{GLC},\text{max}}$, $K_{\text{M},\text{GLC}}$, $v_{\text{XYL},\text{max}}$, and $K_{\text{M},\text{XYL}}$. Equation (A1l) imposes an upper limit for the overall uptake rate of carbon sources with parameter $v_{\text{upt,max}}$. Values for the kinetic parameters were derived from single batch experiments with *C. glutamicum* ${\text{WMB2}}_{\text{evo}}$ (optimized for d‐xylose utilization via the Weimberg pathway, see Radek et al., 2017 for more details) which was grown on either d‐glucose or d‐xylose, respectively (data not shown). For the sake of simplicity and considering confidence bounds, the following parameters were used: $v_{\text{upt,max}}=v_{\text{GLC},\text{max}}=4.5\;\text{mmol}\;{\left(g\;X\;\cdot \;h\right)}^{-1}$; $v_{\text{XYL},\text{max}}=4.0\;\text{mmol}\;{\left(g\;X\;\cdot \;h\right)}^{-1}$; $K_{\text{M},\text{GLC}}=K_{\text{M},\text{XYL}}=15$ mM. The upper bound on oxygen uptake rate (Equation (A1m) with parameter $v_{{\text{O}}_{2},\text{max}}$) can be interpreted as an operational degree of freedom that depends on stirring rate, aeration rate and medium (air or pure oxygen). The initial carbon concentration is related to a batch experiment with *C. glutamicum* ${\text{WMB2}}_{\text{evo}}$ on a mixture of 20 g $L^{-1}$ d‐xylose and 10 g $L^{-1}$ d‐glucose (data not shown). By taking the molar masses of $M_{\text{XYL}}=150.13$ $\text{g}\;{\text{mol}}^{-1}$ and $M_{\text{GLC}}=180.16$ $\text{g}\;{\text{mol}}^{-1}$ and the number of C‐atoms (5 and 6, respectively) into consideration, the initial carbon concentration of 1 mol ${\text{L}}^{-1}$ is obtained. The objective function of the embedded optimization problem is maximization of biomass growth (Equation (A1h)).
